# Multiple intestinal hemangioma concurrent with low-grade appendiceal mucinous neoplasm presenting as intussusception—a case report and literature review

**DOI:** 10.1186/s12957-022-02519-z

**Published:** 2022-02-23

**Authors:** Yanhua Yang, Dongmei Jia, Chen Jiang

**Affiliations:** Department of Pathology, Qingdao Municipal Hospital, Qingdao, 266071 China

**Keywords:** Multiple intestinal hemangioma, Low-grade appendiceal mucinous neoplasm, Intussusceptions, Laparotomy, Hemicolectomy

## Abstract

**Background:**

Cases with intussusception caused by either intestinal hemangiomas or appendiceal mucinous neoplasms are extremely rare.

**Case presentation:**

In this study, we reported a 47-year-old male presented with paroxysmal abdominal pain and postprandial bloating for 3 days. CT results indicated a high possibility of secondary intussusception in ascending colon. Histopathology indicated a mixed type of cavernous and capillary hemangioma, combined with low-grade appendiceal mucinous neoplasms (LAMNs) and intestinal obstruction. The patient underwent laparotomy and right hemicolectomy. Finally, the patient was followed up for 4 months with no disease progression.

**Conclusions:**

Rare studies reported the intestine hemangiomas coincided with appendix low-grade mucinous tumor. Its manifestations are not specific, which is a challenge in the preoperative diagnosis. For cases with intussusception that was not observed in time, it may lead to intestinal necrosis and diffuse peritonitis. Additionally, the ruptured mucinous tumor in the appendix may lead to pathogenesis of pseudomyxoma peritonei. Therefore, accurate diagnosis and appropriate surgery-based treatment contribute to the improvement of prognosis and severe outcomes among these patients.

## Background

Gastrointestinal (GI) hemangiomas are uncommon benign vascular tumors that may occur in any part of the GI tract. According to the size of the involved vessels, hemangiomas are histologically classified into cavernous, capillary, or mixed-type tumors. Cavernous type is the most common, while multiple hemangiomas are very rare. Mucinous neoplasm of the appendix is featured by mucinous epithelial proliferation combined with extracellular mucin and pushing tumor margins. According to the 2019 WHO classification of tumors of the digestive system [1–3], appendiceal mucinous neoplasm was defined as hyperplastic polyp, serrated lesions, low-grade appendiceal mucinous neoplasms (LAMNs), high-grade appendiceal mucinous neoplasms (HAMNs), as well as mucinous adenocarcinoma of appendix, which present with or without appendiceal perforation. To our best knowledge, appendiceal mucinous neoplasms and intestinal hemangiomas are rare in clinical practice. Besides, cases with intussusception caused by either intestinal hemangiomas or appendiceal mucinous neoplasms are extremely rare. In this study, we reported a rare case with simultaneous occurrence of LAMNs and diffuse intestinal hemangioma.

## Case presentation

A 47-year-old male patient was admitted to the Emergency Department in our hospital due to paroxysmal abdominal pain and postprandial bloating for 3 days. The pain was centered in the position around the umbilicus. The flatus and defecation were normal. He showed no fever, hematemesis, hematochezia, diarrhea, melena, or weight loss upon admission. CT performed in a local hospital showed intussusception, intestinal effusion, and pneumatosis. There was no remission in the symptoms after conservative treatment. Besides, the abdominal symptoms showed gradual deterioration.

The findings for the laboratory examination were as follows: white cell count, 7.62 × 10^9^/L, neutrophils, 73.1%; hemoglobin, 145.0 g/L; hematocrit, 46.80%; platelet, 356 × 10^9^/L (Table 1).

**Table 1:** Results of the laboratory examination

Items	Results	Normal range
White cell count	7.62 × 10^9^/L	3.5–9.5 × 10^9^/L
Neutrophils	73.1%	40–75%
RBCs	5.16 × 10^9^/L	4.3–5.8 × 10^9^/L
Hemoglobin	145.0 g/L	115–150 g/L
Hematocrit	46.80%	35–45%
Platelet	204 × 10^9^/L	125–350 × 10^9^/L
MCHC	310.0	316–354
Corpuscular volume	46.8%	40–50%
Mean RBC hemoglobin content	28.10	27–34

*RBC* red blood cell, *MCHC* mean corpuscular hemoglobin concentration

**Fig. 1:**
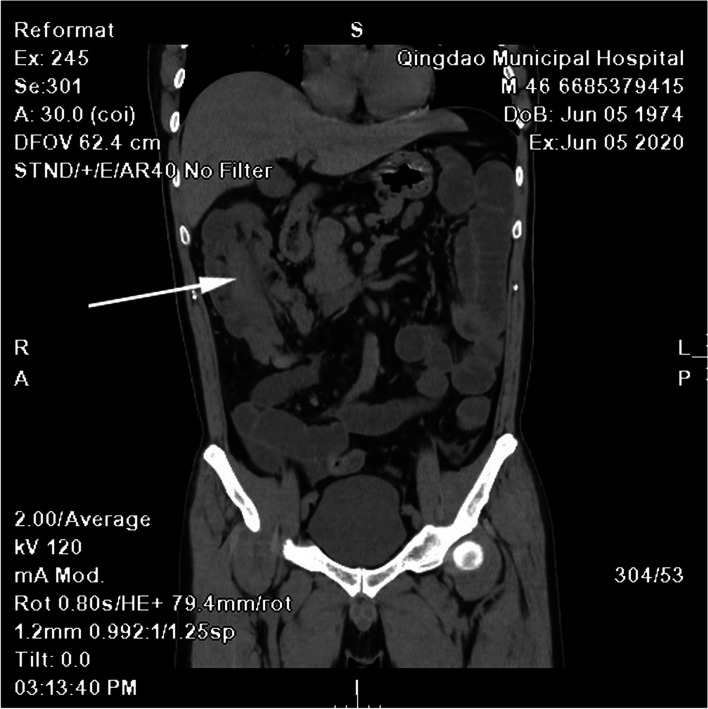
Abdominal computed tomography view indicated ascending colon intussusception (grey arrow)

Abdominal CT scan revealed intussusception in the ascending colon (Fig. 1). The electrocardiogram and chest X-ray findings were normal.

For the treatment, the patient received surgery, in which the skin, subcutaneous tissues, anterior and posterior rectus sheath, and peritoneum were cut in turn. There was no abdominal distension or liquid overflow. The small intestine was slightly swollen and jasmine-colored ascites (500 ml) was seen in the pelvic cavity. The distal ileum, appendix, cecum, and greater omentum were found to be intussuscepted into the ascending colon. The length of the intussuscepted ileum was about 15 cm. The root of appendix and cecal wall was hard, and there was purulent substance on the surface. The greater omentum showed adhesion to the cecal wall. There was slight enlargement in the lymph nodes of radix of mesentery. In addition, adhesion was seen in the lateral region of peritoneum.

**Fig. 2:**
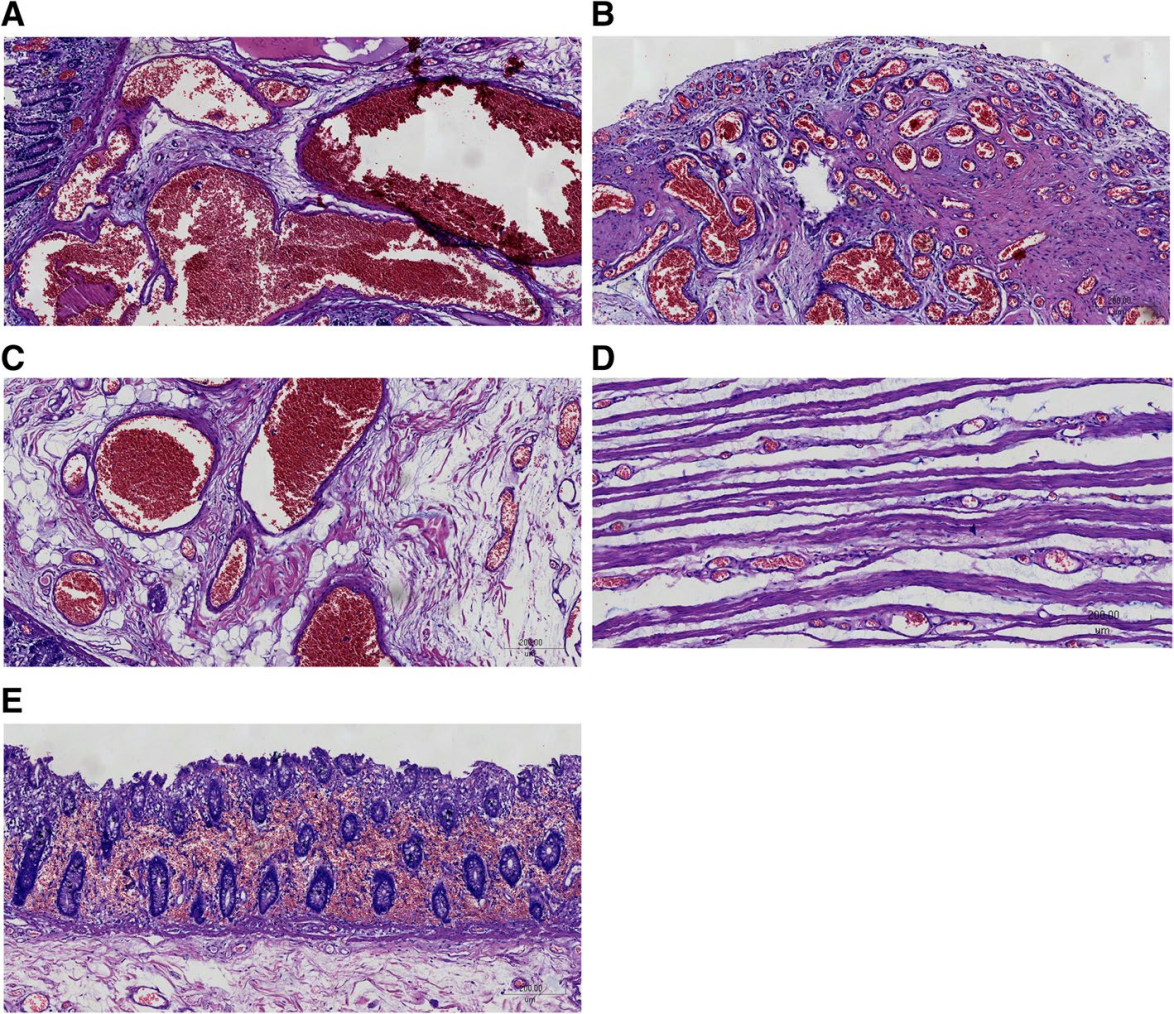
The lesion was unencapsulated and composed of blood-filled spaces of variable size and shape. The lumens were filled with blood cells and lined by thin endothelial cells and separated by connective tissue stroma. The images were obtained after H&E staining under a magnification of 100×. **A**, **B** The lesion of distal ileum stated at submucosa or serosa. **C**, **D** The lesion of colon stated at submucosa or through the muscularis. **E** Some colon mucosa showed interstitial hemorrhage

**Fig. 3:**
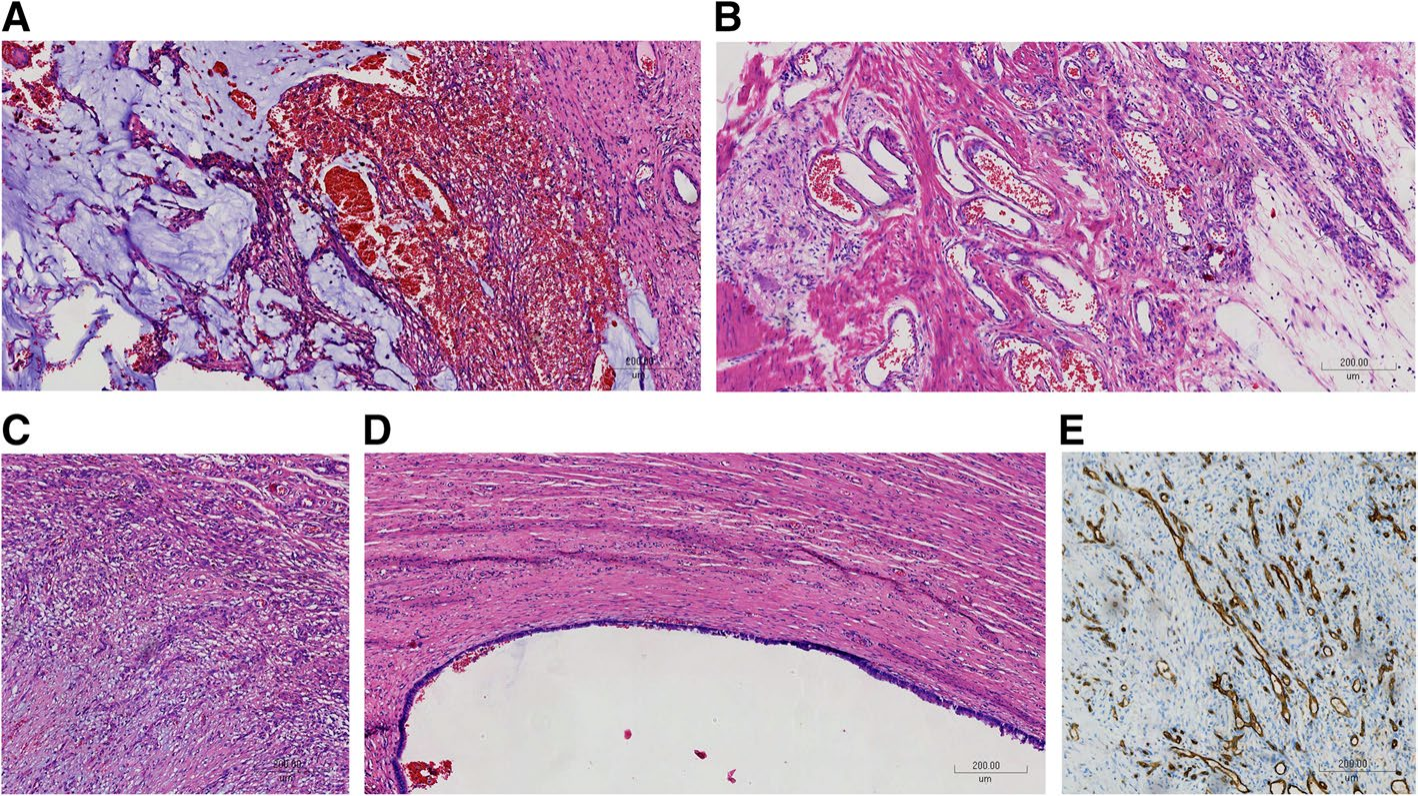
Lesions of appendix with mixed capillary and cavernous hemangiomas and LAM. The images were obtained after H&E staining under a magnification of 100×. **A** There was massive mucus, blood filled parenchymal vessels and hemorrhage in the appendiceal mucosa. **B** Spongy blood vessels of variable size and shape were seen, together with capillaries at the appendix muscularis and serosa. **C** Massive capillaries at the serosa. **D** The distended region of the appendix with thinned mucosa was reduced to a single columnar cell layer, with underlying fibrotic submucosa. The columnar cell was minimal atypia. **E** Immunohistochemical staining of CD31

For the histopathological characteristics, the intestinal wall of ileum, cecum, and ascending colon were thickened in a diffuse manner and the thickest part was about 0.8 cm. The texture was hard and tough, with the serosal surface in a grayish color. Dilated lumen and blood clots were seen in the incisal surface. The mucosal surface of the ileum was erosive, and the colonic was smooth in a grayish-red color. The appendix showed a length of about 5 cm and a diameter of 1.5 cm. The wall of the appendix was hard. Mucoid substance was observed in the appendix cavity near the root. Microscopically, there were blood-filled lumens or cavities in the ileum, cecum, colon submucosa and serosa, which were lined with a single layer of endothelial cells, and the cells were not heteromorphic (Fig. 2A–E). The interstitium was filled with loose fibrous connective tissue, and dilated vascular lumen was found in some muscular layers in smooth muscle bundles. There were proliferative capillaries and large thin-walled vascular lumen in ileum, cecum, mesentery of colon, and serous surface of appendix. Meanwhile, remarkable proliferation was noticed in the interstitial collagen fibers. No anastomosis was noticed in the interacted capillaries. The large vascular lumen was filled with red blood cells (RBCs), and the lumen was lined with monolayer endothelial cells of no atypia. The appendix cavity was completely filled in with mucus. In addition, the appendix lamina propria mucosa and muscularis mucosa were not available. Part of submucosa showed fibrosis. The wall of the appendix was lined with low-grade mucinous epithelium (Fig. 3A–E).

CT results indicated a high possibility of secondary intussusception. Histopathology confirmed mixed type of cavernous and capillary hemangioma, combined with LAMNs and intestinal obstruction. For the treatment, the patient underwent laparotomy and right hemicolectomy. The postoperative recovery was good without any complications. The patient was followed up for 4 months, and no discomforts were reported by the patient until now.

## Discussion

GI hemangiomas refer to a rare condition, with a prevalence of merely 0.05% [4] of all intestinal neoplasms and 2.8% of all intestinal neoplasms [5]. Grossly, GI hemangiomas could be presented as polypoid and intraluminal, or submucosal lesions with diffused or vague boundaries. Most of the lesions were in a color of purplish-red to blue with a soft and compressible texture unless containing a thrombus or phleboliths.

The disease was generally divided into three categories: (i) the capillary hemangioma, described as a small cluster of submucosal capillaries expanded intra-luminally, may develop into a stalk-like mass; (ii) a category represented by mixed capillary and cavernous hemangiomas; and (iii) the cavernous hemangioma, serving as the most common type. The histopathology of our case was mixed capillary and cavernous hemangiomas which involved small intestine, colorectal, and appendix.

The majority of cases with GI hemangiomas presented occult or acute gastrointestinal bleeding signs, followed by bowel obstruction, abdominal pain, perforation, or intussusception. In partial patients, there might be small polypoid lesions incidentally after endoscopy screening. In this case, the patient showed no bleeding or concealed hemorrhage. On this basis, the lesion was not detected in a long time.

Mucinous neoplasm of the appendix was an appendiceal neoplasm characterized by mucinous epithelial proliferation with extracellular mucin and pushing tumor margins. To date, little is known about the etiology of appendiceal mucinous neoplasms. In the 2019 WHO Classification of Tumors of the Digestive System [3, 6], the appendiceal mucinous neoplasm was excluded. According to the epithelial hyperplasia and morphology, the appendiceal mucinous neoplasm was defined as hyperplastic polyp, serrated lesions, LAMNs, HAMNs, and mucinous adenocarcinoma. The appendiceal mucinous cystadenoma was further defined as LAMNs. Diagnostic criteria for LAMNs was villous pseudostratified mucinous epithelium or monolayered mucinous cells with only mild atypia and with a broad pushing margin, fibrosis, hyalinization, and calcification of the appendiceal wall; various degrees of mucin dissection; and the absence of muscularis mucosa.

HAMNs show histological features similar to LAMNs, including subepithelial fibrosis, a broad pushing margin, broad-front pushing invasion, rupture, and peritoneal dissemination. The neoplastic epithelium has unequivocal high-grade features including enlarged, hyperchromatic, and pleomorphic nuclei, numerous atypical mitotic figures, single-cell necrosis, and sloughed necrotic epithelial cells in the lumen of the appendix. In cases of an infiltrative pattern, there would be stromal desmoplasia and mucin pools with atypical cells, as well as content of extracellular mucin > 50% in the lesions. Such condition was defined as mucinous adenocarcinoma.

The clinical symptoms of patients with peritoneal dissemination included progressive abdominal distention, new onset of an umbilical hernia, or a palpable mass after abdominal or pelvic examinations. There might be a soft tissue mass in the appendix that was manifested as hydrops after CT or ultrasonography. To our best knowledge, curvilinear calcification of the wall was the specific lesions, but they were presented in only half of the cases. Our case showed features of hemangiomas of small intestine, cecum and appendix combined with LAMNs.

The prognosis of LAMN is highly depending on the tumor stage [3]. Those with tumors limited to the appendix showed a good prognosis, while those with peritoneal dissemination were reported to show a variable prognosis. The prognosis in disseminated tumors depended on the grade of the peritoneal mucinous epithelium, disease severity, and the ability to achieve complete cytoreduction of macroscopically visible tumor within the abdomen. Hyperthermic intraperitoneal chemotherapy and complete cytoreduction may cause clinical benefits to the patients’ survival. As rare cases are diagnosed with HAMNs, there are limited data regarding their natural history when they are confined to the appendix. Currently, the management of HAMNs was limited to the appendix, and the efficiency of additional surgery is still uncertain. HAMNs patients disseminated to the peritoneal cavity are likely to behave like other mucinous tumors spread to the peritoneum. In our study, the patient was diagnosed with LAMNs with no tumor rupture, and the prognosis was satisfactory in the 4-month follow-up after surgery.

Intussusception is primarily a disease in childhood and is relatively rare in adults. Unlike childhood intussusception, adult intussusception shows identifiable lesions such as malignant or benign neoplasms. However, intussusception caused by hemangioma is a very rare entity. In our literature search, we searched the articles on intestinal hemangioma and intussusceptions published between January 2000 and February 2021 using the following key words: “gastrointestinal hemangioma” and “intussusception”. Finally, 10 articles [6–15] in English with intestinal hemangioma and intussusception were identified. Among these articles, 4 cases [7–10] were about blue rubber bleb nevus syndrome, which was a hereditary cutaneous syndrome characterized by cavernous hemangiomas of skin and GI tract that were thought to represent hamartomas. One case was diagnosed with PHACES syndrome, a newly defined rare congenital disease of hemangioma combined with other organ deformity, which was associated with intestinal hemangioma causing recurrent intussusceptions [6]. Specifically, one case [11] was child and two [12, 13] were teenagers. Only two cases [14, 15] of adult hemangioma were described (Table 2).

**Table 2:** Cases diagnosed with intestinal hemangioma and intussusception reported between January 2000 and February 2021

Author	Patient		Presentations	Hemangioma location	Histology	Intussusception location	Preoperative diagnosis	Treatment
	Sex	Age, year						
Wang et al [1]	M	19	BRBNS with melena and abdominal pain	Jejunum and gastric lumen	Cavernous hemangiomas	Jejunojejunal intussusception	CT	Exploratory, laparotomy, intraoperative esophagogastroduodenoscopy, wedge gastrectomy and a part of jejunum were resected
Menegozzo et al [2]	F	25	Chronic intestinal bleeding presented with acute onset of colic pain and vomit	Small and large bowels, Liver	Cavernous hemangiomas	Jejunum	US, CT	Laparotomy, simple reduction of the intussusception segments
Tzoufi et al [3]	M	13	Complex partial generalized seizures, cerebral palsy, multiple skeletal anomalies anaemia	Skins, mucous membranes, gastrointestinal tract, left occipital lobe	Cavernous hemangiomas	Ileocolic intussusception	CT, MRI	Surgery, blood transfusions
Lee et al [4]	M	37	Colicky abdominal pain, nausea, vomiting	Jejunum, mid-ileum and distal ileum, Bluish skin lesions around neck, chest and abdomen, hepatic, right quadratus lumborum muscle	Cavernous hemangiomas	Jejunum, mid-ileum and distal ileum	Abdominal radiograph, CT	Urgent laparotomy and bowel resections, limited right hemicolectomy
Keiko et al [5]	M	2 years and 10 months	Vomiting, diarrhea, abdominal pain, watery diarrhea with strawberry jelly-like stools	Cecum	Capillary	Colon	US, CT, colonoscopy, barium enema	laparotomy with ileocecal resection and side to-end ileo-ascending colon anastomosis
Khalil et al [6]	F	17	Abdominal pain, lower gastrointestinal bleeding and anaemia	Rectosigmoid, hepatic flexure, small bowel	Multiple cavernous haemangioma	Small bowel	Colonoscopy, nuclear medicine GI bleeding scintigraphic scanning, CT angiogram	Surgical exploration, Enterotomy small bowel resection, colotomy, ligation and excision of the hepatic flexure and rectosigmoid region
Zorica et al [7]	F	13	Vomiting, colicky abdominal pain and constipation.	Ileal	Pyogenic granuloma, lobular capillary hemangioma	ileum	US, Plain abdominal radiography	Laparotomy. The affected gangrenous loop of the small bowel was resected and a temporary terminal ileostomy was performed
Morgan et al [8]	F	21	Abdominal pain and vomiting	Small bowel, lymph nodes	Mixed haemangioma	mid small bowel	US, CT, Gastrography enema	Laparotomy. The affected loop of small bowel was resected, together with the mass and the lymph nodes, and an end to end anastomosis was performed
Kye et al [9]	F	81	Intermittent melena, nausea, dizziness, severe anemia	Jejunum	Polypoid hemangioma of a proliferation of dilated vessels and small capillaries	Jejunum	CT, esophagogastroduodenoscopy colonoscopy (EGD), colonoscopic	Explorative laparotomy, segmental resection of the small bowel
Al-Musali B et al [10]	F	0.25	Projectile vomiting, abdominal pain, and redcurrant jelly stool	Ilium	Diffuse infantile hemangioma	Not available	Laparoscopy, US, CT, MRI, MRA	Surgical resection of the thickened circumferential hemangioma covering the mid ilium along with oral propranolol

*US* ultrasound, *CT* computer tomography, *MRI* magnetic resonance imaging, *MRA* magnetic resonance angiography

Clinically, GI hemangiomas are symptomatic in 90% of cases, unlike other benign tumors of the GI tract that were symptom-free. The most frequent sign was chronic GI bleeding, which caused anemia of an unknown origin and massive bleeding under a rare condition. Occasionally, these tumors may cause intestinal obstructions, intussusception, intramural hematoma, perforation, and platelet sequestration. In a previous study, Fu et al. [16] reported that only a small part of patients (4%) showed shock and intestinal obstructions caused by gastrointestinal hemangioma. In our case, the routine blood examination findings including RBCs, hemoglobin, hematocrit, mean corpuscular volume, and average RBC hemoglobin content, were all in normal ranges. The mean corpuscular hemoglobin concentration was slightly lower than normal range. The fecal occult blood test findings were all negative. There were no bleeding-related symptoms and signs. Among the 10 patients with intussusception obtained after literature search, abdominal pain was the main clinical symptom in 8 (80.0%) patients, followed by nausea or 7 vomiting (70.0%), and 5 intestinal bleeding (50.0%). Based on the histological examinations, 5 cases were confirmed with cavernous hemangioma, 2 with capillary hemangioma, and 2 case with mixed capillary and cavernous hemangiomas, and 1 case with diffuse infantile hemangioma.

Histologically, our case was diagnosed with mixed capillary and cavernous hemangiomas. Indeed, rare adult cases showed intussusception caused by multiple hemangiomas. To our best knowledge, rare cases showed hemangiomas of small intestine, accompanied by large intestine and appendix.

Appendiceal intussusception is a rare condition presenting as acute appendicitis. It is still a challenge in the clinical diagnosis. In a previous study by Collins et al., the authors conducted a 40-year prospective study involving 71,000 cases underwent appendectomy, which indicated an incidence of 0.01% for the appendiceal intussusception [17]. As previously described, the appendiceal intussusception is usually induced by irregular appendiceal peristalsis developed by local irritation, which is more likely to occur in cases with mobile mesoappendix, enlarged appendicular lumen, and thin appendicular wall [18].

In our case, there were a large amount of mucus in the lumen of appendix and abundant dilated capillaries in the mucinous interstitium and the wall of the appendix. Besides, neoplastic hyperplasia of capillaries was seen on the serous surface. The patient was finally diagnosed with mixed hemangioma of the appendix combined with low-grade mucinous tumor. Based on the literature search using the term “appendiceal mucinous tumor” and “intussusception” from the articles in the PubMed database between January 2000 and February 2021, 29 articles [19–47] were obtained, among which 20 [19–21, 23, 24, 26–40] were eligible after excluding 9 articles including 2 [22, 47] in Japanese language and 1[25] in Dutch, 2 [41, 42] involved the cases with no appendiceal mucinous tumor, 2 [44, 46] with appendiceal mucinous tumor with no intussusception, 1 [45] with sigmoidorectal intussusception caused by a mucinous adenocarcinoma of the sigmoid colon, as well as 1 study [43] focused on the histological and imaging features. Among the remaining 20 articles, one article [26] mentioned 3 related cases in the literature review including 1 with LAMNs induced intussusception, 1 with intussusception caused by appendiceal mucocele [48], and 1 with intussusception caused by appendiceal mucocele combined with schistosomiasis [49]. Mucocele was non-neoplastic mucinous lesion characterized by markedly thinned or denuded mucosa combined with luminal dilatation, without atypia and hyperplasia. In addition, one article concerning the clinicopathologic features of 21 cases of inverted appendix reporting 3 cases met the demands of the literature search [40]. Finally, 25 cases were included (Table 3). Then these cases were re-evaluated according to the classification standard in 2019 [1, 3]; among these cases, 18 (72.0%) were confirmed with LAMNs pTis. Specifically, one case was diagnosed LAMNs pTis with rectal medium-low differentiated adenocarcinoma [30]. One case was diagnosed LAMNs pTis associated with ovarian cyst of the follicular-luteinic type [27]. One case (4.0%) was diagnosed with simple retention cysts [49], one (4.0%) with simple retention cysts and Schistosomiasis [50], one (4.0%) with serrated dysplasia of low grade [40], one (4.0%) with LAMNs pT3 [23], two (8.0%) with LAMNs pT4a [32, 38], as well as one (4.0%) with HAMNs of the appendix and well-differentiated neuroendocrine carcinoma [33]. For the classification of intussusception, 8 cases (36.2%) were appendix intussuscepting into the caecum, and 15 cases (60.0%) were colonic intussusception. In addition, one case showed Colo-colic intussusception with secondary ileocolic intussusception. One case showed distal ileum and appendix intussuscepted into the cecum.

**Table 3:** Cases with appendiceal mucinous tumor and intussusception reported between January 2000 and February 2021

Author	Patient		Presentation	Histology	Intussusception location	Preoperative diagnosis study	Treatment
	Sex	Age					
Chua et al [1]	F	30	Worsening right lower abdominal pain associated with nausea, vomiting, diarrhea	LAMN pTis	Ileocolic Intussusception	CT	Partial caecectomy with appendicectomy
Houlzé-Laroye [2]	F	35	Abdominal symptoms evoking an intestinal obstruction	LAMN pTis	Ileo-caecal intussusception (ileo-caecal intussusception through the left transverse colon)	CT	Exploratory laparotomy and bowel resection
Nakamatsu et al [3]	F	43	Right lower quadrant pain	LAMN pTis	Colonic intussusception located in the transverse colon	Contrast-enhanced CT, biopsy colonoscopy	Elective laparoscopy assisted ileocecal resection with lymph node dissection
Oliphant et al [4]	F	20	Colicky central abdominal pain, vomiting, constipation	LAMN pT3	Ileocolic Intussusception	CT	Extended right hemicolectomy
Coulier et al [5]		40	Intermittent right abdominal pain accompanied by a palpable mass in the right flank	LAMN pTis	Colo-colic intussusception with secondary ileocolic intussusception	Helical CT, US	Surgical reduction of the intussuscepting complex and ileo-cecal
Feliu et al [6]	F	57	Intermittent and worsening abdominal pain, dizziness, diarrhea	LAMN pTis	Ileocolic intussusception	X-ray, CT, laparoscopic	Right hemicolectomy
Cois et al [7]	F	36	Colicky pain located in the central abdomen, radiating to the flanks and associated with nausea	LAMN pTis	Right colonic intussusception	CT, US	Laparotomy, partial resection of the caecum and right ovary
Yamaguchi et al [8]	F	32	Intermittent abdominal pain and nonbloody diarrhea	LAMN pTis	Colic intussusception	X-ray, CT, colonoscopy, biopsy, barium enema	Laparoscopic reduction of intussusception and ileocecal resection
Rudek et al [9]	M	52	acute right lower abdominal pain	LAMN pTis	Appendix was partially intussuscepted into the cecum	Plain X-rays, US, CT	Ileocecal resection with regional lymphadenectomy, ileocolic anastomosis
Sun et al [10]	F	57	Hematochezia, changes in defecation habits, mild swelling and pain in the right lower quadrant of abdomen	LAMN pTis and rectal medium-low differentiated adenocarcinoma (cT4N1M0)	Appendix intussuscepted into the Cecum	CT, colonoscopy, biopsy	Resection of both the appendix and ileocecum, radical resection of the rectal Carcinoma, regular chemotherapy for rectal carcinoma
Okuda et al [11]	M	44	Asymptomatic	LAMN pTis	Ileocolic intussusception	US, reconstructed CT images, coronal and sagittal multiplanar reconstruction (MPR) images, MRI	Laparoscopic ileocecectomy
Lin et al [12]	M	28	Diffuse abdominal pain but more localized over the epigastric area and right lower quadrant with McBurney point tenderness	LAMN pT4a	Ileocolic intussusception	CT scan	Right hemicolectomy
Butte et al [13]	F	41	Episodic abdominal pain, abdominal distention and nausea	HAMN and well-differentiated neuroendocrine carcinoma	Distal ileum and appendix intussuscepted into the cecum	US, colonoscopy, CT, Biopsies	Right colectomy
Waseem et al [14]	F	84	Caecum intussuscepting into the transverse colon along with the appendix and ileum	LAMN pTis	Caecum intussuscepting into the transverse colon along with the appendix and ileum ileocaeco-colic intussusception	Plain radiographs, US	forceful reduction, Right hemicolectomy and ileocolic anastomosis
Teke et al [15]	F	37	Acute onset, right lower abdominal pain	LAMN pTis	Appendix intussuscepting into the caecum	CT	Ileocaecal resection
Laalim et al [16]	F	47	pain in the right side of the abdomen, nausea and vomiting	LAMN pTis	Appendiceal intussusception to the cecum and secondary distal ileum invagination	US, CT	Laparoscopic right hemicolectomy
Davey MG et al [17]	M	42	Sudden onset abdominal pain and bloody diarrhea	LAMN pTis	ileocecal intussusception at the hepatic flexure	Endoscopy, CT	Right hemicolectomy
Ashrafifi M et al [18]	M	35	Abdominal pain and an altered bowel habit., diarrhea, vomiting, and abdominal distension, anorexia	LAMN pT4a	Appendiceal intussusception to the cecum	Digital rectal examination and rigid sigmoidoscopy Colonoscopy, Biopsies, CT colonography	Appendicectomy, partial caecectomy
Blondiaux E et al [19]	F	63	Right lower quadrant abdominal pain	Simple retention cysts	Dilated proximal portion of the appendix intussuscepted in the cecum lumen	CT	Right hemi-colectomy
Siddiqi AJ et al [20]	F	22	Acute right lower quadrant pain	LAMN pTis	Ileocolic intussusception with an appendiceal mucocele serving as the lead point	Multidetector CT scan of the abdomen and pelvis	Appendectomy and ileocecal resection
Wong MT et al [21]	F	81	Intermittent right-sided abdominal pain	Simple retention cysts and Schistosomiasis	colonic intussusception	Digital rectal examination, plain abdominal radiographs, CT	Right hemicolectomy
Chan Ket et al [22]	F	41	Intermittent, right-sided abdominal pain, abdominal distension, nausea, and vomiting	LAMN pTis	Ileocolic intussusception	CT, Colonoscopy	Laparoscopic right hemicolectomy
Birkness J et al [23]	F	46	–	LAMN pTis	Appendiceal intussusception	Colonoscopy	Right colon resection
Birkness J et al [23]	F	64	–	LAMN pTis	Appendiceal intussusception	Colonoscopy	Cecal resection
Birkness J et al [23]	F	47	–	Serrated dysplasia, Low grade	Appendiceal intussusception	Colonoscopy	Right colon resection

*US* ultrasound, *CT* computer tomography, − data not available, *MRI* magnetic resonance imaging, *MRA* magnetic resonance angiography, *LAMN* low-grade appendiceal mucinous neoplasms, *HAMN* high-grade appendiceal mucinous neoplasm

LAMN pTis: LAMN confined to the muscularis propria (defined as involvement by acellular mucin or mucinous epithelium that may extend into muscularis propria)

LAMN pT3: LAMNs invades subserosa or mesoappendix

LAMN pT4a: LAMNs perforates visceral peritoneum, including mucinous peritoneal tumor or acellular mucin on the serosa of the appendix or mesoappendix

LAMN pT4b: LAMNs directly invades other organs or structures

Appendiceal mucinous tumor and intestinal hemangioma were considered to lead to intussusception based on literature review. Intussusception caused by appendiceal mucinous tumor is more common than that caused by adult hemangioma. In our case, a large amount of mucus was observed in the appendix cavity, and there was more mucus in the residual mucosa interstitium of the appendix. Meanwhile, capillary proliferation was seen in the mucous background. A large number of new neovascularization was found in the submucosa, muscular layer and serosa surface of ileum, as well as cecum appendix and colon. We speculated that appendiceal mucinous tumor may lead to stiffness of intestinal wall, and disorder of peristalsis rhythm. On this basis, it may affect the blood circulation of local tissues, and then the congestion and telangiectasia would result in aggravation in the formation of new capillaries. In cases of hemangioma of colon and ileocecal region, hemangioma would greatly increase the load of intestinal peristalsis, which is likely to induce dysfunction of intestinal movement. Therefore, it may induce the accumulation of appendix mucus, which led to a possibility of occurrence and/or progression of low-grade mucinous tumors of the appendix. It has been well acknowledged that both appendiceal mucinous tumor and intestinal diffuse hemangioma are chronic processes. No matter which showed onset first or collision, the disorder of intestinal blood supplies and mucinous tumor all can lead to the disorder of intestinal movement rhythm. Mucinous tumor and hemangioma contributed to their mutual progression, which eventually resulted in intussusception. Pathological analysis indicated no necrosis in the colon wall. Besides, only erosion of the epithelium and the hemorrhage of the lamina propria were seen on the mucosal surface, which further confirmed that the occurrence of intussusception in our case was a chronic process. In the 4-month follow-up, the symptoms of gastrointestinal disorders showed attenuation with no significant postoperative complication.

## Conclusion

Multiple hemangiomas of small and large intestine are rare in adults. Rare studies reported the intestine hemangiomas coincided with appendix low-grade mucinous tumor. In clinical settings, the manifestations were not specific, which was a challenge in the preoperative diagnosis. For cases with intussusception that was not observed in time, it may lead to intestinal necrosis and diffuse peritonitis. In this study, we described a rare case with intussusception caused by either intestinal hemangiomas or appendiceal mucinous neoplasms. We described a possibility of intussusception. In addition, contrast-enhanced CT scan clearly showed hemangioma and mucous lesions. In this study, the patient underwent plain CT scan, which confirmed the presence of intussusception. On this basis, exploratory laparotomy and surgery were conducted. In the presence of confirmed diagnosis, the surgery should be given.

## Data Availability

The datasets used and analyzed during the current study are available from the corresponding author on reasonable request.

## Abbreviations

GI: Gastrointestinal
LAMNS: Low-grade appendiceal mucinous neoplasms
HAMNS: High-grade appendiceal mucinous neoplasms
